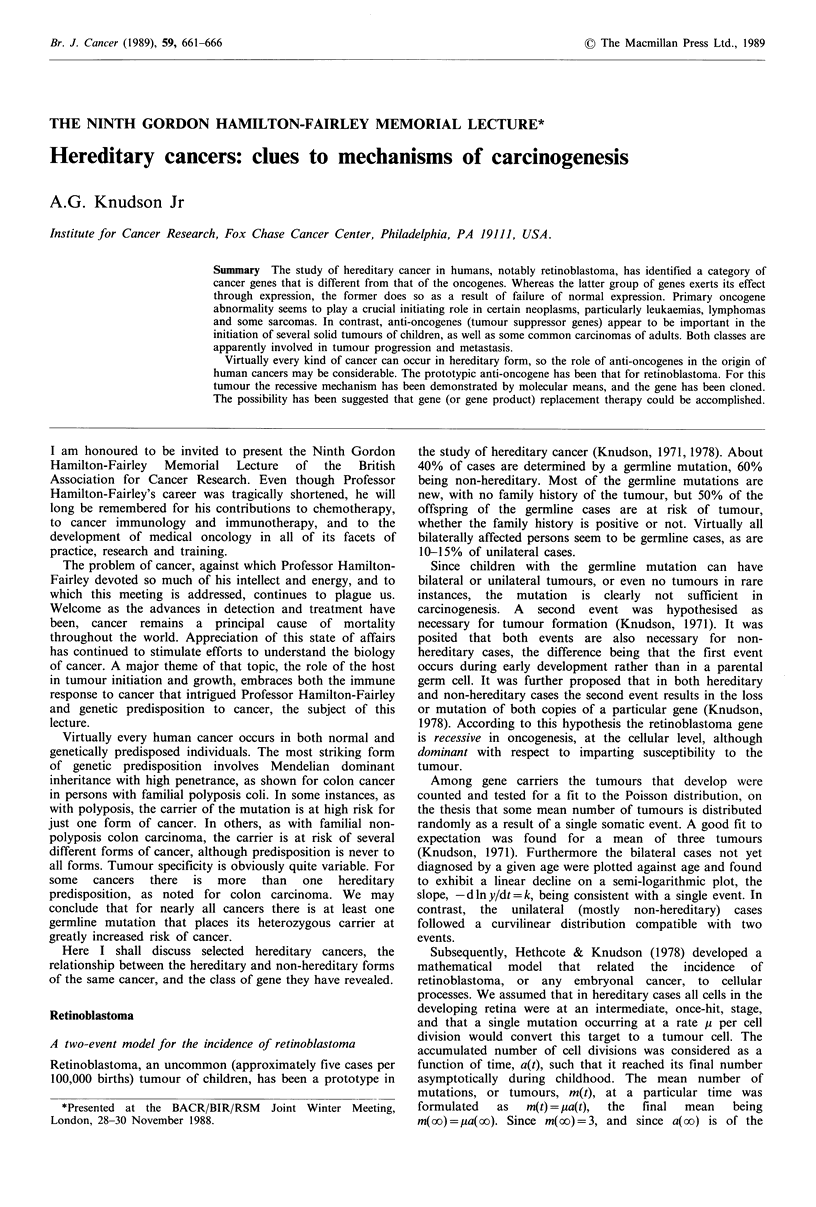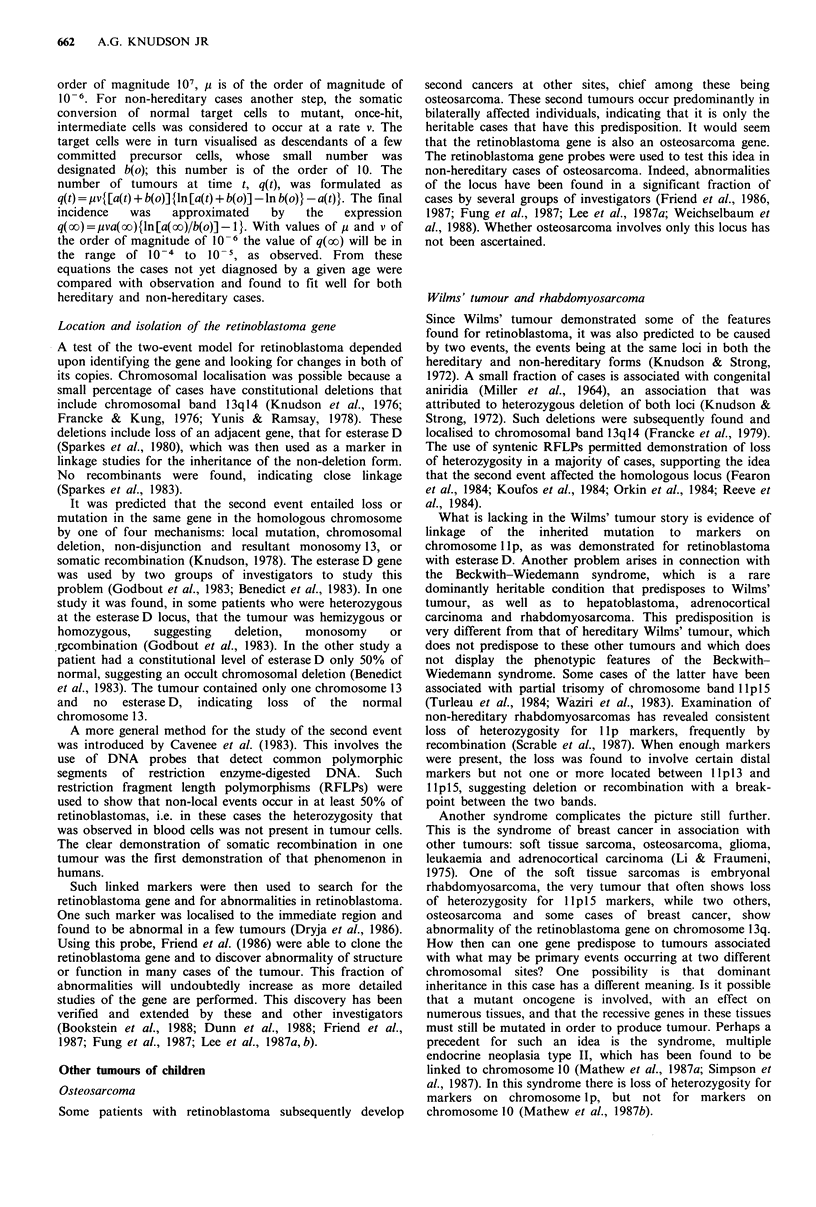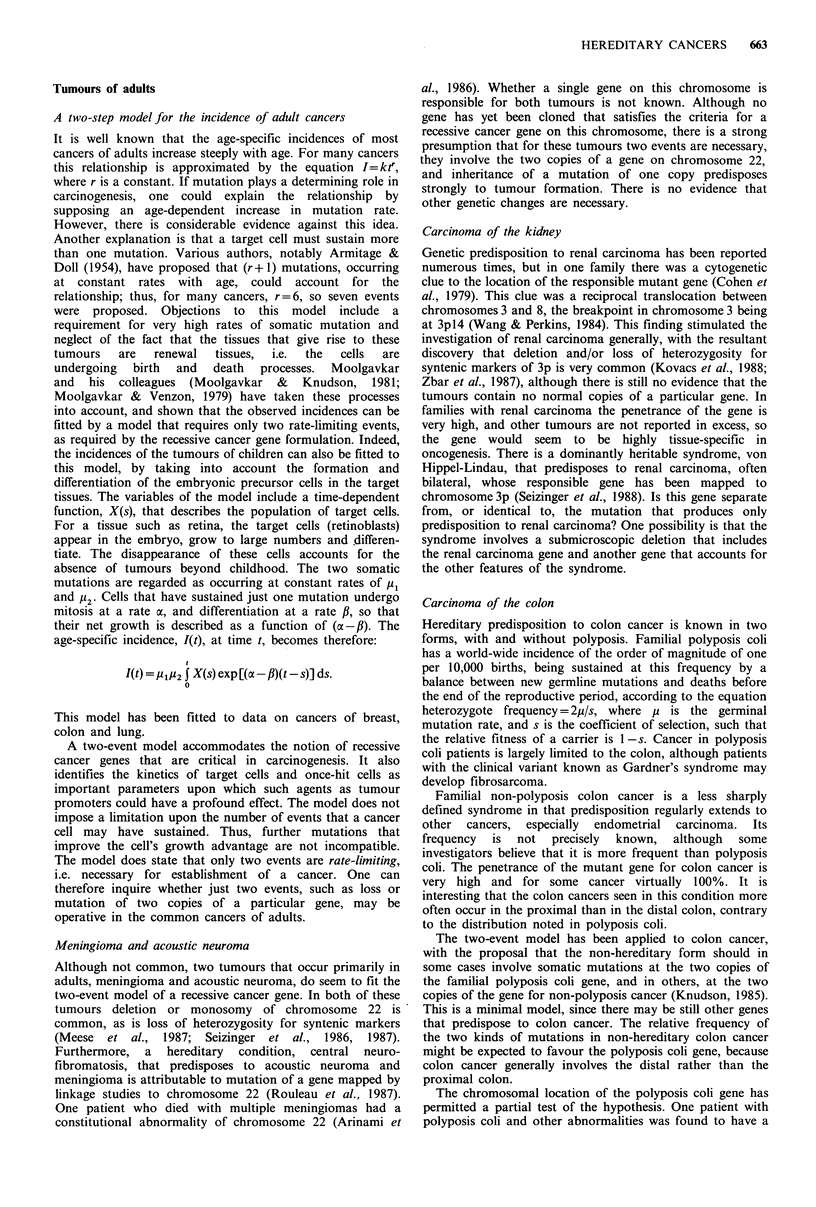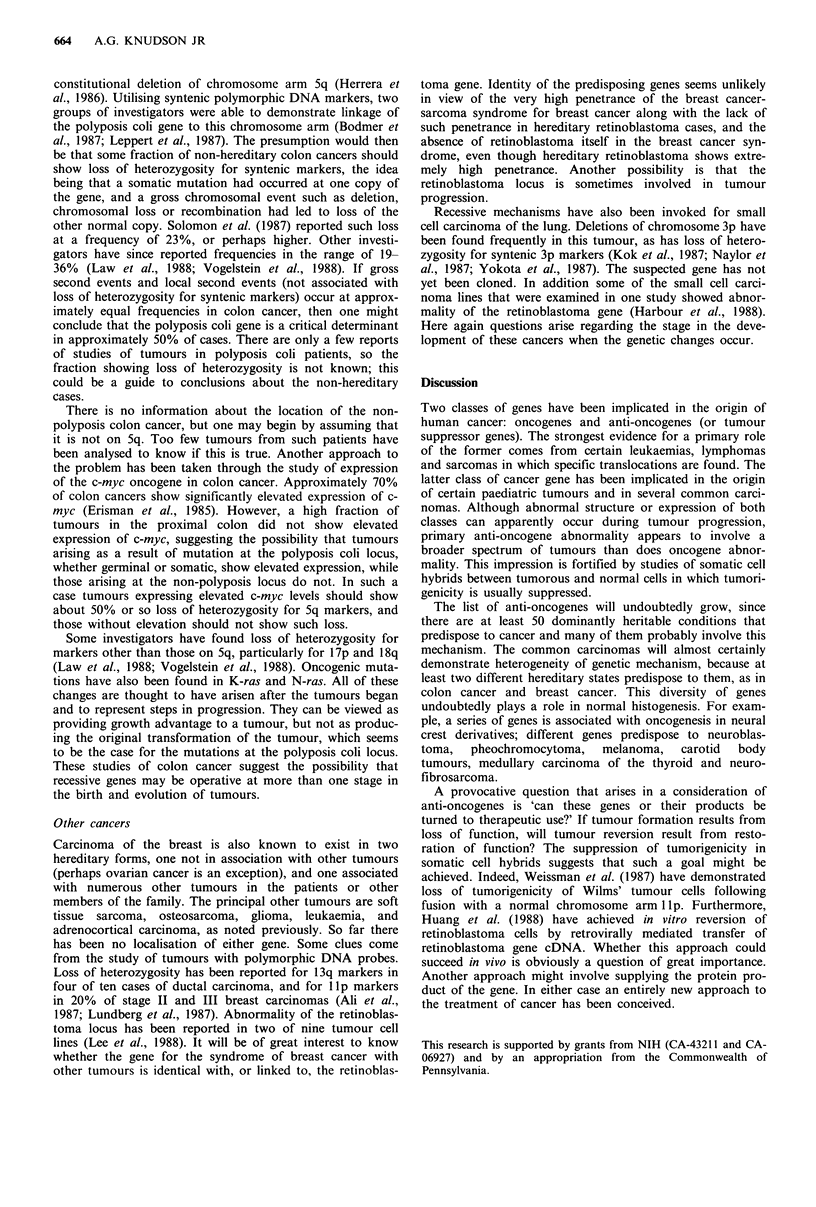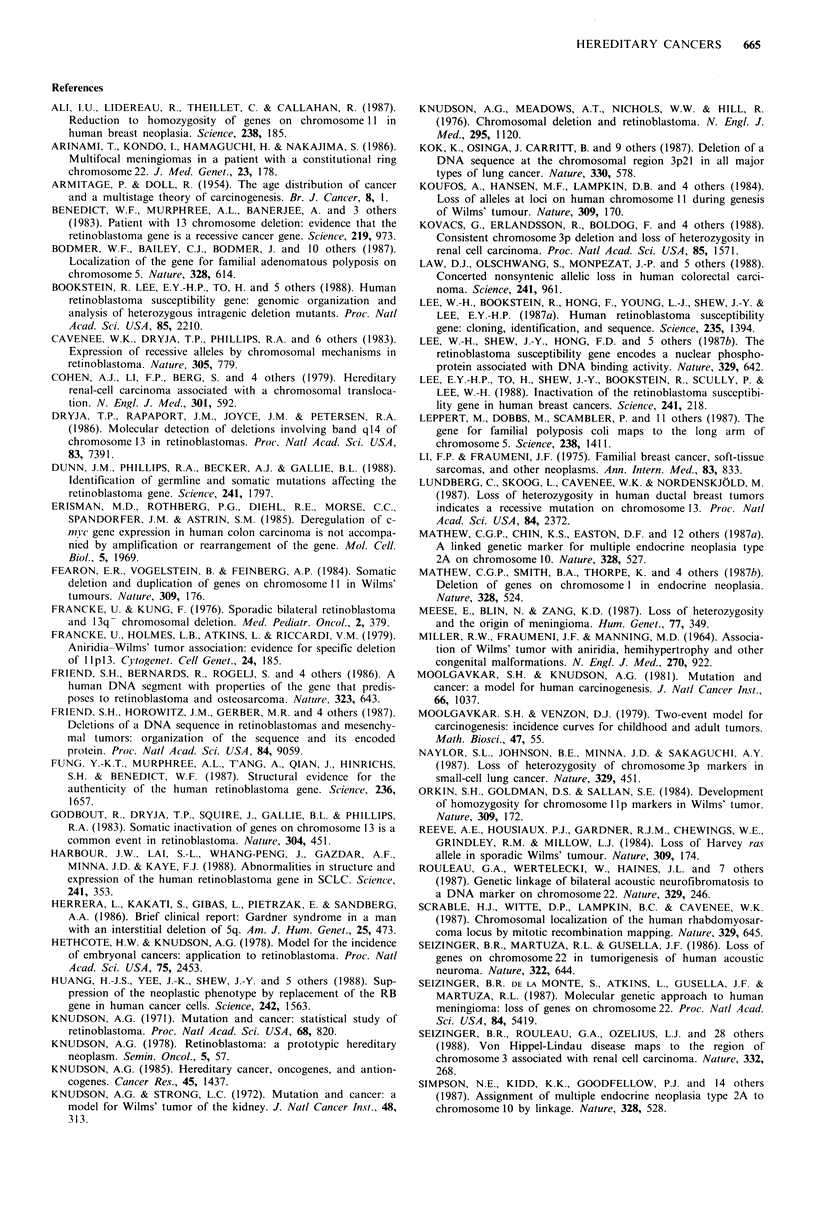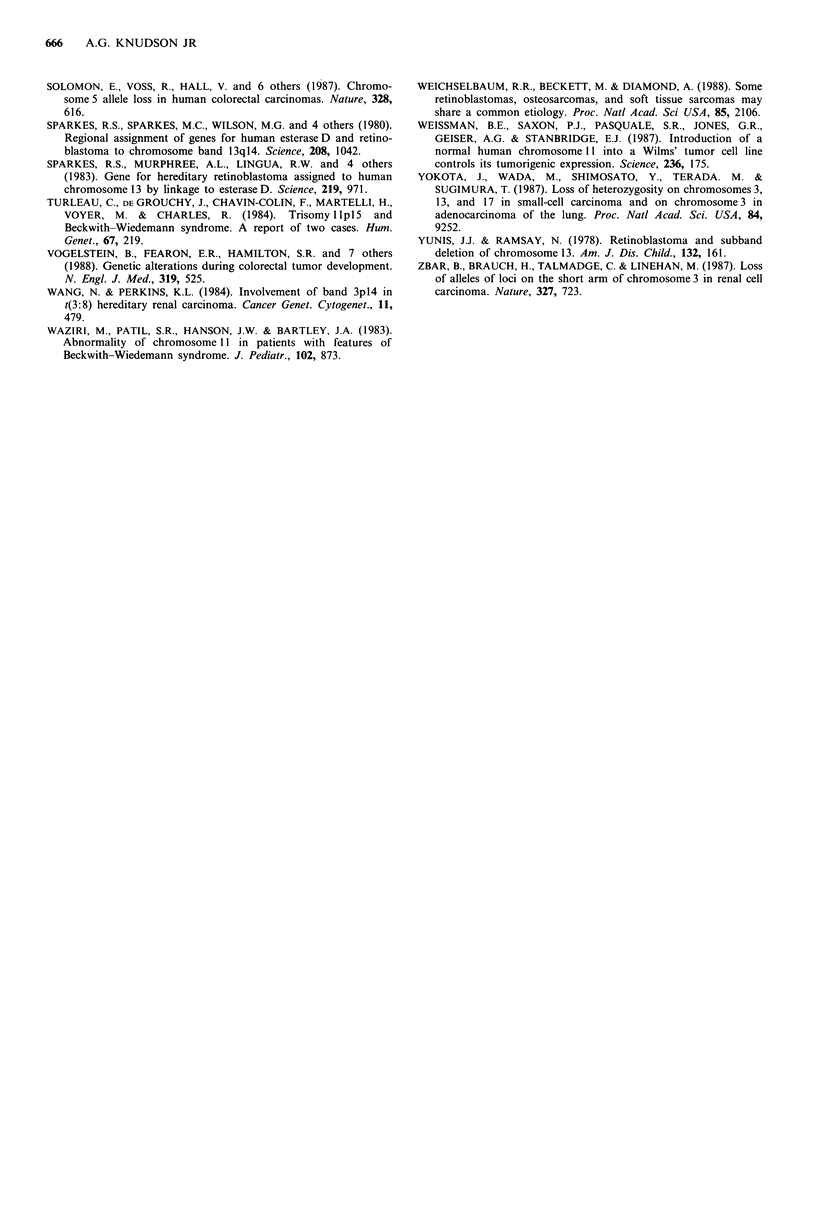# The ninth Gordon Hamilton-Fairley memorial lecture. Hereditary cancers: clues to mechanisms of carcinogenesis.

**DOI:** 10.1038/bjc.1989.137

**Published:** 1989-05

**Authors:** A. G. Knudson

**Affiliations:** Institute for Cancer Research, Fox Chase Cancer Center, Philadelphia, PA 19111.

## Abstract

The study of hereditary cancer in humans, notably retinoblastoma, has identified a category of cancer genes that is different from that of the oncogenes. Whereas the latter group of genes exerts its effect through expression, the former does so as a result of failure of normal expression. Primary oncogene abnormality seems to play a crucial initiating role in certain neoplasms, particularly leukaemias, lymphomas and some sarcomas. In contrast, anti-oncogenes (tumour suppressor genes) appear to be important in the initiation of several solid tumours of children, as well as some common carcinomas of adults. Both classes are apparently involved in tumour progression and metastasis. Virtually every kind of cancer can occur in hereditary form, so the role of anti-oncogenes in the origin of human cancers may be considerable. The prototypic anti-oncogene has been that for retinoblastoma. For this tumour the recessive mechanism has been demonstrated by molecular means, and the gene has been cloned. The possibility has been suggested that gene (or gene product) replacement therapy could be accomplished.


*Br. J. Cancer* (1989), **59**, 661–666 © The Macmillan Press Ltd., 1989

### THE NINTH GORDON HAMILTON-FAIRLEY MEMORIAL LECTURE*

### Hereditary cancers: clues to mechanisms of carcinogenesis

A.G. Knudson Jr

*Institute for Cancer Research, Fox Chase Cancer Center, Philadelphia, PA 19111, USA.*

**Summary** The study of hereditary cancer in humans, notably retinoblastoma, has identified a category of cancer genes that is different from that of the oncogenes. Whereas the latter group of genes exerts its effect through expression, the former does so as a result of failure of normal expression. Primary oncogene abnormality seems to play a crucial initiating role in certain neoplasms, particularly leukaemias, lymphomas and some sarcomas. In contrast, anti-oncogenes (tumour suppressor genes) appear to be important in the initiation of several solid tumours of children, as well as some common carcinomas of adults. Both classes are apparently involved in tumour progression and metastasis.

Virtually every kind of cancer can occur in hereditary form, so the role of anti-oncogenes in the origin of human cancers may be considerable. The prototypic anti-oncogene has been that for retinoblastoma. For this tumour the recessive mechanism has been demonstrated by molecular means, and the gene has been cloned. The possibility has been suggested that gene (or gene product) replacement therapy could be accomplished.

I am honoured to be invited to present the Ninth Gordon Hamilton-Fairley Memorial Lecture of the British Association for Cancer Research. Even though Professor Hamilton-Fairley's career was tragically shortened, he will long be remembered for his contributions to chemotherapy, to cancer immunology and immunotherapy, and to the development of medical oncology in all of its facets of practice, research and training.

The problem of cancer, against which Professor Hamilton-Fairley devoted so much of his intellect and energy, and to which this meeting is addressed, continues to plague us. Welcome as the advances in detection and treatment have been, cancer remains a principal cause of mortality throughout the world. Appreciation of this state of affairs has continued to stimulate efforts to understand the biology of cancer. A major theme of that topic, the role of the host in tumour initiation and growth, embraces both the immune response to cancer that intrigued Professor Hamilton-Fairley and genetic predisposition to cancer, the subject of this lecture.

Virtually every human cancer occurs in both normal and genetically predisposed individuals. The most striking form of genetic predisposition involves Mendelian dominant inheritance with high penetrance, as shown for colon cancer in persons with familial polyposis coli. In some instances, as with polyposis, the carrier of the mutation is at high risk for just one form of cancer. In others, as with familial non-polyposis colon carcinoma, the carrier is at risk of several different forms of cancer, although predisposition is never to all forms. Tumour specificity is obviously quite variable. For some cancers there is more than one hereditary predisposition, as noted for colon carcinoma. We may conclude that for nearly all cancers there is at least one germline mutation that places its heterozygous carrier at greatly increased risk of cancer.

Here I shall discuss selected hereditary cancers, the relationship between the hereditary and non-hereditary forms of the same cancer, and the class of gene they have revealed.

### Retinoblastoma

*A two-event model for the incidence of retinoblastoma*

Retinoblastoma, an uncommon (approximately five cases per 100,000 births) tumour of children, has been a prototype in the study of hereditary cancer (Knudson, 1971, 1978). About 40% of cases are determined by a germline mutation, 60% being non-hereditary. Most of the germline mutations are new, with no family history of the tumour, but 50% of the offspring of the germline cases are at risk of tumour, whether the family history is positive or not. Virtually all bilaterally affected persons seem to be germline cases, as are 10–15% of unilateral cases.

Since children with the germline mutation can have bilateral or unilateral tumours, or even no tumours in rare instances, the mutation is clearly not sufficient in carcinogenesis. A second event was hypothesised as necessary for tumour formation (Knudson, 1971). It was posited that both events are also necessary for non-hereditary cases, the difference being that the first event occurs during early development rather than in a parental germ cell. It was further proposed that in both hereditary and non-hereditary cases the second event results in the loss or mutation of both copies of a particular gene (Knudson, 1978). According to this hypothesis the retinoblastoma gene is *recessive* in oncogenesis, at the cellular level, although *dominant* with respect to imparting susceptibility to the tumour.

Among gene carriers the tumours that develop were counted and tested for a fit to the Poisson distribution, on the thesis that some mean number of tumours is distributed randomly as a result of a single somatic event. A good fit to expectation was found for a mean of three tumours (Knudson, 1971). Furthermore the bilateral cases not yet diagnosed by a given age were plotted against age and found to exhibit a linear decline on a semi-logarithmic plot, the slope, $-\mathrm{d}\ln y/\mathrm{d}t = k$, being consistent with a single event. In contrast, the unilateral (mostly non-hereditary) cases followed a curvilinear distribution compatible with two events.

Subsequently, Hethcote & Knudson (1978) developed a mathematical model that related the incidence of retinoblastoma, or any embryonal cancer, to cellular processes. We assumed that in hereditary cases all cells in the developing retina were at an intermediate, once-hit, stage, and that a single mutation occurring at a rate $\mu$ per cell division would convert this target to a tumour cell. The accumulated number of cell divisions was considered as a function of time, $a(t)$, such that it reached its final number asymptotically during childhood. The mean number of mutations, or tumours, $m(t)$, at a particular time was formulated as $m(t) = \mu a(t)$, the final mean being $m(\infty) = \mu a(\infty)$. Since $m(\infty) = 3$, and since $a(\infty)$ is of the

*Presented at the BACR/BIR/RSM Joint Winter Meeting, London, 28–30 November 1988.

order of magnitude $10^7$, $\mu$ is of the order of magnitude of $10^{-6}$. For non-hereditary cases another step, the somatic conversion of normal target cells to mutant, once-hit, intermediate cells was considered to occur at a rate $v$. The target cells were in turn visualised as descendants of a few committed precursor cells, whose small number was designated $b(o)$; this number is of the order of 10. The number of tumours at time $t$, $q(t)$, was formulated as $q(t) = \mu v\{[a(t) + b(o)]\{\ln[a(t) + b(o)] - \ln b(o)\} - a(t)\}$. The final incidence was approximated by the expression $q(\infty) = \mu v a(\infty)\{\ln[a(\infty)/b(o)] - 1\}$. With values of $\mu$ and $v$ of the order of magnitude of $10^{-6}$ the value of $q(\infty)$ will be in the range of $10^{-4}$ to $10^{-5}$, as observed. From these equations the cases not yet diagnosed by a given age were compared with observation and found to fit well for both hereditary and non-hereditary cases.

*Location and isolation of the retinoblastoma gene*

A test of the two-event model for retinoblastoma depended upon identifying the gene and looking for changes in both of its copies. Chromosomal localisation was possible because a small percentage of cases have constitutional deletions that include chromosomal band 13q14 (Knudson *et al.*, 1976; Francke & Kung, 1976; Yunis & Ramsay, 1978). These deletions include loss of an adjacent gene, that for esterase D (Sparkes *et al.*, 1980), which was then used as a marker in linkage studies for the inheritance of the non-deletion form. No recombinants were found, indicating close linkage (Sparkes *et al.*, 1983).

It was predicted that the second event entailed loss or mutation in the same gene in the homologous chromosome by one of four mechanisms: local mutation, chromosomal deletion, non-disjunction and resultant monosomy 13, or somatic recombination (Knudson, 1978). The esterase D gene was used by two groups of investigators to study this problem (Godbout *et al.*, 1983; Benedict *et al.*, 1983). In one study it was found, in some patients who were heterozygous at the esterase D locus, that the tumour was hemizygous or homozygous, suggesting deletion, monosomy or recombination (Godbout *et al.*, 1983). In the other study a patient had a constitutional level of esterase D only 50% of normal, suggesting an occult chromosomal deletion (Benedict *et al.*, 1983). The tumour contained only one chromosome 13 and no esterase D, indicating loss of the normal chromosome 13.

A more general method for the study of the second event was introduced by Cavenee *et al.* (1983). This involves the use of DNA probes that detect common polymorphic segments of restriction enzyme-digested DNA. Such restriction fragment length polymorphisms (RFLPs) were used to show that non-local events occur in at least 50% of retinoblastomas, i.e. in these cases the heterozygosity that was observed in blood cells was not present in tumour cells. The clear demonstration of somatic recombination in one tumour was the first demonstration of that phenomenon in humans.

Such linked markers were then used to search for the retinoblastoma gene and for abnormalities in retinoblastoma. One such marker was localised to the immediate region and found to be abnormal in a few tumours (Dryja *et al.*, 1986). Using this probe, Friend *et al.* (1986) were able to clone the retinoblastoma gene and to discover abnormality of structure or function in many cases of the tumour. This fraction of abnormalities will undoubtedly increase as more detailed studies of the gene are performed. This discovery has been verified and extended by these and other investigators (Bookstein *et al.*, 1988; Dunn *et al.*, 1988; Friend *et al.*, 1987; Fung *et al.*, 1987; Lee *et al.*, 1987*a*, *b*).

### Other tumours of children

*Osteosarcoma*

Some patients with retinoblastoma subsequently develop second cancers at other sites, chief among these being osteosarcoma. These second tumours occur predominantly in bilaterally affected individuals, indicating that it is only the heritable cases that have this predisposition. It would seem that the retinoblastoma gene is also an osteosarcoma gene. The retinoblastoma gene probes were used to test this idea in non-hereditary cases of osteosarcoma. Indeed, abnormalities of the locus have been found in a significant fraction of cases by several groups of investigators (Friend *et al.*, 1986, 1987; Fung *et al.*, 1987; Lee *et al.*, 1987*a*; Weichselbaum *et al.*, 1988). Whether osteosarcoma involves only this locus has not been ascertained.

*Wilms' tumour and rhabdomyosarcoma*

Since Wilms' tumour demonstrated some of the features found for retinoblastoma, it was also predicted to be caused by two events, the events being at the same loci in both the hereditary and non-hereditary forms (Knudson & Strong, 1972). A small fraction of cases is associated with congenital aniridia (Miller *et al.*, 1964), an association that was attributed to heterozygous deletion of both loci (Knudson & Strong, 1972). Such deletions were subsequently found and localised to chromosomal band 13q14 (Francke *et al.*, 1979). The use of syntenic RFLPs permitted demonstration of loss of heterozygosity in a majority of cases, supporting the idea that the second event affected the homologous locus (Fearon *et al.*, 1984; Koufos *et al.*, 1984; Orkin *et al.*, 1984; Reeve *et al.*, 1984).

What is lacking in the Wilms' tumour story is evidence of linkage of the inherited mutation to markers on chromosome 11p, as was demonstrated for retinoblastoma with esterase D. Another problem arises in connection with the Beckwith–Wiedemann syndrome, which is a rare dominantly heritable condition that predisposes to Wilms' tumour, as well as to hepatoblastoma, adrenocortical carcinoma and rhabdomyosarcoma. This predisposition is very different from that of hereditary Wilms' tumour, which does not predispose to these other tumours and which does not display the phenotypic features of the Beckwith–Wiedemann syndrome. Some cases of the latter have been associated with partial trisomy of chromosome band 11p15 (Turleau *et al.*, 1984; Waziri *et al.*, 1983). Examination of non-hereditary rhabdomyosarcomas has revealed consistent loss of heterozygosity for 11p markers, frequently by recombination (Scrable *et al.*, 1987). When enough markers were present, the loss was found to involve certain distal markers but not one or more located between 11p13 and 11p15, suggesting deletion or recombination with a breakpoint between the two bands.

Another syndrome complicates the picture still further. This is the syndrome of breast cancer in association with other tumours: soft tissue sarcoma, osteosarcoma, glioma, leukaemia and adrenocortical carcinoma (Li & Fraumeni, 1975). One of the soft tissue sarcomas is embryonal rhabdomyosarcoma, the very tumour that often shows loss of heterozygosity for 11p15 markers, while two others, osteosarcoma and some cases of breast cancer, show abnormality of the retinoblastoma gene on chromosome 13q. How then can one gene predispose to tumours associated with what may be primary events occurring at two different chromosomal sites? One possibility is that dominant inheritance in this case has a different meaning. Is it possible that a mutant oncogene is involved, with an effect on numerous tissues, and that the recessive genes in these tissues must still be mutated in order to produce tumour. Perhaps a precedent for such an idea is the syndrome, multiple endocrine neoplasia type II, which has been found to be linked to chromosome 10 (Mathew *et al.*, 1987*a*; Simpson *et al.*, 1987). In this syndrome there is loss of heterozygosity for markers on chromosome 1p, but not for markers on chromosome 10 (Mathew *et al.*, 1987*b*).

### Tumours of adults

### *A two-step model for the incidence of adult cancers*

It is well known that the age-specific incidences of most cancers of adults increase steeply with age. For many cancers this relationship is approximated by the equation $I = kt^r$, where $r$ is a constant. If mutation plays a determining role in carcinogenesis, one could explain the relationship by supposing an age-dependent increase in mutation rate. However, there is considerable evidence against this idea. Another explanation is that a target cell must sustain more than one mutation. Various authors, notably Armitage & Doll (1954), have proposed that $(r+1)$ mutations, occurring at constant rates with age, could account for the relationship; thus, for many cancers, $r=6$, so seven events were proposed. Objections to this model include a requirement for very high rates of somatic mutation and neglect of the fact that the tissues that give rise to these tumours are renewal tissues, i.e. the cells are undergoing birth and death processes. Moolgavkar and his colleagues (Moolgavkar & Knudson, 1981; Moolgavkar & Venzon, 1979) have taken these processes into account, and shown that the observed incidences can be fitted by a model that requires only two rate-limiting events, as required by the recessive cancer gene formulation. Indeed, the incidences of the tumours of children can also be fitted to this model, by taking into account the formation and differentiation of the embryonic precursor cells in the target tissues. The variables of the model include a time-dependent function, $X(s)$, that describes the population of target cells. For a tissue such as retina, the target cells (retinoblasts) appear in the embryo, grow to large numbers and differentiate. The disappearance of these cells accounts for the absence of tumours beyond childhood. The two somatic mutations are regarded as occurring at constant rates of $\mu_1$ and $\mu_2$. Cells that have sustained just one mutation undergo mitosis at a rate $\alpha$, and differentiation at a rate $\beta$, so that their net growth is described as a function of $(\alpha-\beta)$. The age-specific incidence, $I(t)$, at time $t$, becomes therefore:

$$I(t) = \mu_1\mu_2 \int_0^t X(s) \exp[(\alpha-\beta)(t-s)]\, ds.$$

This model has been fitted to data on cancers of breast, colon and lung.

A two-event model accommodates the notion of recessive cancer genes that are critical in carcinogenesis. It also identifies the kinetics of target cells and once-hit cells as important parameters upon which such agents as tumour promoters could have a profound effect. The model does not impose a limitation upon the number of events that a cancer cell may have sustained. Thus, further mutations that improve the cell's growth advantage are not incompatible. The model does state that only two events are *rate-limiting*, i.e. necessary for establishment of a cancer. One can therefore inquire whether just two events, such as loss or mutation of two copies of a particular gene, may be operative in the common cancers of adults.

### *Meningioma and acoustic neuroma*

Although not common, two tumours that occur primarily in adults, meningioma and acoustic neuroma, do seem to fit the two-event model of a recessive cancer gene. In both of these tumours deletion or monosomy of chromosome 22 is common, as is loss of heterozygosity for syntenic markers (Meese *et al.*, 1987; Seizinger *et al.*, 1986, 1987). Furthermore, a hereditary condition, central neurofibromatosis, that predisposes to acoustic neuroma and meningioma is attributable to mutation of a gene mapped by linkage studies to chromosome 22 (Rouleau *et al.*, 1987). One patient who died with multiple meningiomas had a constitutional abnormality of chromosome 22 (Arinami *et al.*, 1986). Whether a single gene on this chromosome is responsible for both tumours is not known. Although no gene has yet been cloned that satisfies the criteria for a recessive cancer gene on this chromosome, there is a strong presumption that for these tumours two events are necessary, they involve the two copies of a gene on chromosome 22, and inheritance of a mutation of one copy predisposes strongly to tumour formation. There is no evidence that other genetic changes are necessary.

### *Carcinoma of the kidney*

Genetic predisposition to renal carcinoma has been reported numerous times, but in one family there was a cytogenetic clue to the location of the responsible mutant gene (Cohen *et al.*, 1979). This clue was a reciprocal translocation between chromosomes 3 and 8, the breakpoint in chromosome 3 being at 3p14 (Wang & Perkins, 1984). This finding stimulated the investigation of renal carcinoma generally, with the resultant discovery that deletion and/or loss of heterozygosity for syntenic markers of 3p is very common (Kovacs *et al.*, 1988; Zbar *et al.*, 1987), although there is still no evidence that the tumours contain no normal copies of a particular gene. In families with renal carcinoma the penetrance of the gene is very high, and other tumours are not reported in excess, so the gene would seem to be highly tissue-specific in oncogenesis. There is a dominantly heritable syndrome, von Hippel-Lindau, that predisposes to renal carcinoma, often bilateral, whose responsible gene has been mapped to chromosome 3p (Seizinger *et al.*, 1988). Is this gene separate from, or identical to, the mutation that produces only predisposition to renal carcinoma? One possibility is that the syndrome involves a submicroscopic deletion that includes the renal carcinoma gene and another gene that accounts for the other features of the syndrome.

### *Carcinoma of the colon*

Hereditary predisposition to colon cancer is known in two forms, with and without polyposis. Familial polyposis coli has a world-wide incidence of the order of magnitude of one per 10,000 births, being sustained at this frequency by a balance between new germline mutations and deaths before the end of the reproductive period, according to the equation heterozygote frequency $= 2\mu/s$, where $\mu$ is the germinal mutation rate, and $s$ is the coefficient of selection, such that the relative fitness of a carrier is $1-s$. Cancer in polyposis coli patients is largely limited to the colon, although patients with the clinical variant known as Gardner's syndrome may develop fibrosarcoma.

Familial non-polyposis colon cancer is a less sharply defined syndrome in that predisposition regularly extends to other cancers, especially endometrial carcinoma. Its frequency is not precisely known, although some investigators believe that it is more frequent than polyposis coli. The penetrance of the mutant gene for colon cancer is very high and for some cancer virtually 100%. It is interesting that the colon cancers seen in this condition more often occur in the proximal than in the distal colon, contrary to the distribution noted in polyposis coli.

The two-event model has been applied to colon cancer, with the proposal that the non-hereditary form should in some cases involve somatic mutations at the two copies of the familial polyposis coli gene, and in others, at the two copies of the gene for non-polyposis cancer (Knudson, 1985). This is a minimal model, since there may be still other genes that predispose to colon cancer. The relative frequency of the two kinds of mutations in non-hereditary colon cancer might be expected to favour the polyposis coli gene, because colon cancer generally involves the distal rather than the proximal colon.

The chromosomal location of the polyposis coli gene has permitted a partial test of the hypothesis. One patient with polyposis coli and other abnormalities was found to have a constitutional deletion of chromosome arm 5q (Herrera *et al.*, 1986). Utilising syntenic polymorphic DNA markers, two groups of investigators were able to demonstrate linkage of the polyposis coli gene to this chromosome arm (Bodmer *et al.*, 1987; Leppert *et al.*, 1987). The presumption would then be that some fraction of non-hereditary colon cancers should show loss of heterozygosity for syntenic markers, the idea being that a somatic mutation had occurred at one copy of the gene, and a gross chromosomal event such as deletion, chromosomal loss or recombination had led to loss of the other normal copy. Solomon *et al.* (1987) reported such loss at a frequency of 23%, or perhaps higher. Other investigators have since reported frequencies in the range of 19–36% (Law *et al.*, 1988; Vogelstein *et al.*, 1988). If gross second events and local second events (not associated with loss of heterozygosity for syntenic markers) occur at approximately equal frequencies in colon cancer, then one might conclude that the polyposis coli gene is a critical determinant in approximately 50% of cases. There are only a few reports of studies of tumours in polyposis coli patients, so the fraction showing loss of heterozygosity is not known; this could be a guide to conclusions about the non-hereditary cases.

There is no information about the location of the non-polyposis colon cancer, but one may begin by assuming that it is not on 5q. Too few tumours from such patients have been analysed to know if this is true. Another approach to the problem has been taken through the study of expression of the c-*myc* oncogene in colon cancer. Approximately 70% of colon cancers show significantly elevated expression of c-*myc* (Erisman *et al.*, 1985). However, a high fraction of tumours in the proximal colon did not show elevated expression of c-*myc*, suggesting the possibility that tumours arising as a result of mutation at the polyposis coli locus, whether germinal or somatic, show elevated expression, while those arising at the non-polyposis locus do not. In such a case tumours expressing elevated c-*myc* levels should show about 50% or so loss of heterozygosity for 5q markers, and those without elevation should not show such loss.

Some investigators have found loss of heterozygosity for markers other than those on 5q, particularly for 17p and 18q (Law *et al.*, 1988; Vogelstein *et al.*, 1988). Oncogenic mutations have also been found in K-*ras* and N-*ras*. All of these changes are thought to have arisen after the tumours began and to represent steps in progression. They can be viewed as providing growth advantage to a tumour, but not as producing the original transformation of the tumour, which seems to be the case for the mutations at the polyposis coli locus. These studies of colon cancer suggest the possibility that recessive genes may be operative at more than one stage in the birth and evolution of tumours.

### *Other cancers*

Carcinoma of the breast is also known to exist in two hereditary forms, one not in association with other tumours (perhaps ovarian cancer is an exception), and one associated with numerous other tumours in the patients or other members of the family. The principal other tumours are soft tissue sarcoma, osteosarcoma, glioma, leukaemia, and adrenocortical carcinoma, as noted previously. So far there has been no localisation of either gene. Some clues come from the study of tumours with polymorphic DNA probes. Loss of heterozygosity has been reported for 13q markers in four of ten cases of ductal carcinoma, and for 11p markers in 20% of stage II and III breast carcinomas (Ali *et al.*, 1987; Lundberg *et al.*, 1987). Abnormality of the retinoblastoma locus has been reported in two of nine tumour cell lines (Lee *et al.*, 1988). It will be of great interest to know whether the gene for the syndrome of breast cancer with other tumours is identical with, or linked to, the retinoblastoma gene. Identity of the predisposing genes seems unlikely in view of the very high penetrance of the breast cancer-sarcoma syndrome for breast cancer along with the lack of such penetrance in hereditary retinoblastoma cases, and the absence of retinoblastoma itself in the breast cancer syndrome, even though hereditary retinoblastoma shows extremely high penetrance. Another possibility is that the retinoblastoma locus is sometimes involved in tumour progression.

Recessive mechanisms have also been invoked for small cell carcinoma of the lung. Deletions of chromosome 3p have been found frequently in this tumour, as has loss of heterozygosity for syntenic 3p markers (Kok *et al.*, 1987; Naylor *et al.*, 1987; Yokota *et al.*, 1987). The suspected gene has not yet been cloned. In addition some of the small cell carcinoma lines that were examined in one study showed abnormality of the retinoblastoma gene (Harbour *et al.*, 1988). Here again questions arise regarding the stage in the development of these cancers when the genetic changes occur.

### Discussion

Two classes of genes have been implicated in the origin of human cancer: oncogenes and anti-oncogenes (or tumour suppressor genes). The strongest evidence for a primary role of the former comes from certain leukaemias, lymphomas and sarcomas in which specific translocations are found. The latter class of cancer gene has been implicated in the origin of certain paediatric tumours and in several common carcinomas. Although abnormal structure or expression of both classes can apparently occur during tumour progression, primary anti-oncogene abnormality appears to involve a broader spectrum of tumours than does oncogene abnormality. This impression is fortified by studies of somatic cell hybrids between tumorous and normal cells in which tumorigenicity is usually suppressed.

The list of anti-oncogenes will undoubtedly grow, since there are at least 50 dominantly heritable conditions that predispose to cancer and many of them probably involve this mechanism. The common carcinomas will almost certainly demonstrate heterogeneity of genetic mechanism, because at least two different hereditary states predispose to them, as in colon cancer and breast cancer. This diversity of genes undoubtedly plays a role in normal histogenesis. For example, a series of genes is associated with oncogenesis in neural crest derivatives; different genes predispose to neuroblastoma, pheochromocytoma, melanoma, carotid body tumours, medullary carcinoma of the thyroid and neurofibrosarcoma.

A provocative question that arises in a consideration of anti-oncogenes is 'can these genes or their products be turned to therapeutic use?' If tumour formation results from loss of function, will tumour reversion result from restoration of function? The suppression of tumorigenicity in somatic cell hybrids suggests that such a goal might be achieved. Indeed, Weissman *et al.* (1987) have demonstrated loss of tumorigenicity of Wilms' tumour cells following fusion with a normal chromosome arm 11p. Furthermore, Huang *et al.* (1988) have achieved *in vitro* reversion of retinoblastoma cells by retrovirally mediated transfer of retinoblastoma gene cDNA. Whether this approach could succeed *in vivo* is obviously a question of great importance. Another approach might involve supplying the protein product of the gene. In either case an entirely new approach to the treatment of cancer has been conceived.

This research is supported by grants from NIH (CA-43211 and CA-06927) and by an appropriation from the Commonwealth of Pennsylvania.

### References

ALI, I.U., LIDEREAU, R., THEILLET, C. & CALLAHAN, R. (1987). Reduction to homozygosity of genes on chromosome 11 in human breast neoplasia. *Science*, **238**, 185.

ARINAMI, T., KONDO, I., HAMAGUCHI, H. & NAKAJIMA, S. (1986). Multifocal meningiomas in a patient with a constitutional ring chromosome 22. *J. Med. Genet.*, **23**, 178.

ARMITAGE, P. & DOLL, R. (1954). The age distribution of cancer and a multistage theory of carcinogenesis. *Br. J. Cancer*, **8**, 1.

BENEDICT, W.F., MURPHREE, A.L., BANERJEE, A. and 3 others (1983). Patient with 13 chromosome deletion: evidence that the retinoblastoma gene is a recessive cancer gene. *Science*, **219**, 973.

BODMER, W.F., BAILEY, C.J., BODMER, J. and 10 others (1987). Localization of the gene for familial adenomatous polyposis on chromosome 5. *Nature*, **328**, 614.

BOOKSTEIN, R. LEE, E.Y.-H.P., TO, H. and 5 others (1988). Human retinoblastoma susceptibility gene: genomic organization and analysis of heterozygous intragenic deletion mutants. *Proc. Natl Acad. Sci. USA*, **85**, 2210.

CAVENEE, W.K., DRYJA, T.P., PHILLIPS, R.A. and 6 others (1983). Expression of recessive alleles by chromosomal mechanisms in retinoblastoma. *Nature*, **305**, 779.

COHEN, A.J., LI, F.P., BERG, S. and 4 others (1979). Hereditary renal-cell carcinoma associated with a chromosomal translocation. *N. Engl. J. Med.*, **301**, 592.

DRYJA, T.P., RAPAPORT, J.M., JOYCE, J.M. & PETERSEN, R.A. (1986). Molecular detection of deletions involving band q14 of chromosome 13 in retinoblastomas. *Proc. Natl Acad. Sci. USA*, **83**, 7391.

DUNN, J.M., PHILLIPS, R.A., BECKER, A.J. & GALLIE, B.L. (1988). Identification of germline and somatic mutations affecting the retinoblastoma gene. *Science*, **241**, 1797.

ERISMAN, M.D., ROTHBERG, P.G., DIEHL, R.E., MORSE, C.C., SPANDORFER, J.M. & ASTRIN, S.M. (1985). Deregulation of c-*myc* gene expression in human colon carcinoma is not accompanied by amplification or rearrangement of the gene. *Mol. Cell. Biol.*, **5**, 1969.

FEARON, E.R., VOGELSTEIN, B. & FEINBERG, A.P. (1984). Somatic deletion and duplication of genes on chromosome 11 in Wilms' tumours. *Nature*, **309**, 176.

FRANCKE, U. & KUNG, F. (1976). Sporadic bilateral retinoblastoma and 13q$^-$ chromosomal deletion. *Med. Pediatr. Oncol.*, **2**, 379.

FRANCKE, U., HOLMES, L.B., ATKINS, L. & RICCARDI, V.M. (1979). Aniridia–Wilms' tumor association: evidence for specific deletion of 11p13. *Cytogenet. Cell Genet.*, **24**, 185.

FRIEND, S.H., BERNARDS, R., ROGELJ, S. and 4 others (1986). A human DNA segment with properties of the gene that predisposes to retinoblastoma and osteosarcoma. *Nature*, **323**, 643.

FRIEND, S.H., HOROWITZ, J.M., GERBER, M.R. and 4 others (1987). Deletions of a DNA sequence in retinoblastomas and mesenchymal tumors: organization of the sequence and its encoded protein. *Proc. Natl Acad. Sci. USA*, **84**, 9059.

FUNG, Y.-K.T., MURPHREE, A.L., T'ANG, A., QIAN, J., HINRICHS, S.H. & BENEDICT, W.F. (1987). Structural evidence for the authenticity of the human retinoblastoma gene. *Science*, **236**, 1657.

GODBOUT, R., DRYJA, T.P., SQUIRE, J., GALLIE, B.L. & PHILLIPS, R.A. (1983). Somatic inactivation of genes on chromosome 13 is a common event in retinoblastoma. *Nature*, **304**, 451.

HARBOUR, J.W., LAI, S.-L., WHANG-PENG, J., GAZDAR, A.F., MINNA, J.D. & KAYE, F.J. (1988). Abnormalities in structure and expression of the human retinoblastoma gene in SCLC. *Science*, **241**, 353.

HERRERA, L., KAKATI, S., GIBAS, L., PIETRZAK, E. & SANDBERG, A.A. (1986). Brief clinical report: Gardner syndrome in a man with an interstitial deletion of 5q. *Am. J. Hum. Genet.*, **25**, 473.

HETHCOTE, H.W. & KNUDSON, A.G. (1978). Model for the incidence of embryonal cancers: application to retinoblastoma. *Proc. Natl Acad. Sci. USA*, **75**, 2453.

HUANG, H.-J.S., YEE, J.-K., SHEW, J.-Y. and 5 others (1988). Suppression of the neoplastic phenotype by replacement of the RB gene in human cancer cells. *Science*, **242**, 1563.

KNUDSON, A.G. (1971). Mutation and cancer: statistical study of retinoblastoma. *Proc. Natl Acad. Sci. USA*, **68**, 820.

KNUDSON, A.G. (1978). Retinoblastoma: a prototypic hereditary neoplasm. *Semin. Oncol.*, **5**, 57.

KNUDSON, A.G. (1985). Hereditary cancer, oncogenes, and antioncogenes. *Cancer Res.*, **45**, 1437.

KNUDSON, A.G. & STRONG, L.C. (1972). Mutation and cancer: a model for Wilms' tumor of the kidney. *J. Natl Cancer Inst.*, **48**, 313.

KNUDSON, A.G., MEADOWS, A.T., NICHOLS, W.W. & HILL, R. (1976). Chromosomal deletion and retinoblastoma. *N. Engl. J. Med.*, **295**, 1120.

KOK, K., OSINGA, J. CARRITT, B. and 9 others (1987). Deletion of a DNA sequence at the chromosomal region 3p21 in all major types of lung cancer. *Nature*, **330**, 578.

KOUFOS, A., HANSEN, M.F., LAMPKIN, D.B. and 4 others (1984). Loss of alleles at loci on human chromosome 11 during genesis of Wilms' tumour. *Nature*, **309**, 170.

KOVACS, G., ERLANDSSON, R., BOLDOG, F. and 4 others (1988). Consistent chromosome 3p deletion and loss of heterozygosity in renal cell carcinoma. *Proc. Natl Acad. Sci. USA*, **85**, 1571.

LAW, D.J., OLSCHWANG, S., MONPEZAT, J.-P. and 5 others (1988). Concerted nonsyntenic allelic loss in human colorectal carcinoma. *Science*, **241**, 961.

LEE, W.-H., BOOKSTEIN, R., HONG, F., YOUNG, L.-J., SHEW, J.-Y. & LEE, E.Y.-H.P. (1987*a*). Human retinoblastoma susceptibility gene: cloning, identification, and sequence. *Science*, **235**, 1394.

LEE, W.-H., SHEW, J.-Y., HONG, F.D. and 5 others (1987*b*). The retinoblastoma susceptibility gene encodes a nuclear phosphoprotein associated with DNA binding activity. *Nature*, **329**, 642.

LEE, E.Y.-H.P., TO, H., SHEW, J.-Y., BOOKSTEIN, R., SCULLY, P. & LEE, W.-H. (1988). Inactivation of the retinoblastoma susceptibility gene in human breast cancers. *Science*, **241**, 218.

LEPPERT, M., DOBBS, M., SCAMBLER, P. and 11 others (1987). The gene for familial polyposis coli maps to the long arm of chromosome 5. *Science*, **238**, 1411.

LI, F.P. & FRAUMENI, J.F. (1975). Familial breast cancer, soft-tissue sarcomas, and other neoplasms. *Ann. Intern. Med.*, **83**, 833.

LUNDBERG, C., SKOOG, L., CAVENEE, W.K. & NORDENSKJÖLD, M. (1987). Loss of heterozygosity in human ductal breast tumors indicates a recessive mutation on chromosome 13. *Proc. Natl Acad. Sci. USA*, **84**, 2372.

MATHEW, C.G.P., CHIN, K.S., EASTON, D.F. and 12 others (1987*a*). A linked genetic marker for multiple endocrine neoplasia type 2A on chromosome 10. *Nature*, **328**, 527.

MATHEW, C.G.P., SMITH, B.A., THORPE, K. and 4 others (1987*b*). Deletion of genes on chromosome 1 in endocrine neoplasia. *Nature*, **328**, 524.

MEESE, E., BLIN, N. & ZANG, K.D. (1987). Loss of heterozygosity and the origin of meningioma. *Hum. Genet.*, **77**, 349.

MILLER, R.W., FRAUMENI, J.F. & MANNING, M.D. (1964). Association of Wilms' tumor with aniridia, hemihypertrophy and other congenital malformations. *N. Engl. J. Med.*, **270**, 922.

MOOLGAVKAR, S.H. & KNUDSON, A.G. (1981). Mutation and cancer: a model for human carcinogenesis. *J. Natl Cancer Inst.*, **66**, 1037.

MOOLGAVKAR. S.H. & VENZON, D.J. (1979). Two-event model for carcinogenesis: incidence curves for childhood and adult tumors. *Math. Biosci.*, **47**, 55.

NAYLOR, S.L., JOHNSON, B.E., MINNA, J.D. & SAKAGUCHI, A.Y. (1987). Loss of heterozygosity of chromosome 3p markers in small-cell lung cancer. *Nature*, **329**, 451.

ORKIN, S.H., GOLDMAN, D.S. & SALLAN, S.E. (1984). Development of homozygosity for chromosome 11p markers in Wilms' tumor. *Nature*, **309**, 172.

REEVE, A.E., HOUSIAUX. P.J., GARDNER, R.J.M., CHEWINGS, W.E., GRINDLEY, R.M. & MILLOW, L.J. (1984). Loss of Harvey *ras* allele in sporadic Wilms' tumour. *Nature*, **309**, 174.

ROULEAU, G.A., WERTELECKI, W., HAINES, J.L. and 7 others (1987). Genetic linkage of bilateral acoustic neurofibromatosis to a DNA marker on chromosome 22. *Nature*, **329**, 246.

SCRABLE, H.J., WITTE, D.P., LAMPKIN, B.C. & CAVENEE, W.K. (1987). Chromosomal localization of the human rhabdomyosarcoma locus by mitotic recombination mapping. *Nature*, **329**, 645.

SEIZINGER, B.R., MARTUZA, R.L. & GUSELLA, J.F. (1986). Loss of genes on chromosome 22 in tumorigenesis of human acoustic neuroma. *Nature*, **322**, 644.

SEIZINGER, B.R. DE LA MONTE, S., ATKINS, L., GUSELLA, J.F. & MARTUZA, R.L. (1987). Molecular genetic approach to human meningioma: loss of genes on chromosome 22. *Proc. Natl Acad. Sci. USA*, **84**, 5419.

SEIZINGER, B.R., ROULEAU, G.A., OZELIUS, L.J. and 28 others (1988). Von Hippel-Lindau disease maps to the region of chromosome 3 associated with renal cell carcinoma. *Nature*, **332**, 268.

SIMPSON, N.E., KIDD, K.K., GOODFELLOW, P.J. and 14 others (1987). Assignment of multiple endocrine neoplasia type 2A to chromosome 10 by linkage. *Nature*, **328**, 528.

SOLOMON, E., VOSS, R., HALL, V. and 6 others (1987). Chromosome 5 allele loss in human colorectal carcinomas. *Nature*, **328**, 616.

SPARKES, R.S., SPARKES, M.C., WILSON, M.G. and 4 others (1980). Regional assignment of genes for human esterase D and retinoblastoma to chromosome band 13q14. *Science*, **208**, 1042.

SPARKES, R.S., MURPHREE, A.L., LINGUA, R.W. and 4 others (1983). Gene for hereditary retinoblastoma assigned to human chromosome 13 by linkage to esterase D. *Science*, **219**, 971.

TURLEAU, C., DE GROUCHY, J., CHAVIN-COLIN, F., MARTELLI, H., VOYER, M. & CHARLES, R. (1984). Trisomy 11p15 and Beckwith–Wiedemann syndrome. A report of two cases. *Hum. Genet.*, **67**, 219.

VOGELSTEIN, B., FEARON, E.R., HAMILTON, S.R. and 7 others (1988). Genetic alterations during colorectal tumor development. *N. Engl. J. Med.*, **319**, 525.

WANG, N. & PERKINS, K.L. (1984). Involvement of band 3p14 in *t*(3:8) hereditary renal carcinoma. *Cancer Genet. Cytogenet.*, **11**, 479.

WAZIRI, M., PATIL, S.R., HANSON, J.W. & BARTLEY, J.A. (1983). Abnormality of chromosome 11 in patients with features of Beckwith–Wiedemann syndrome. *J. Pediatr.*, **102**, 873.

WEICHSELBAUM, R.R., BECKETT, M. & DIAMOND, A. (1988). Some retinoblastomas, osteosarcomas, and soft tissue sarcomas may share a common etiology. *Proc. Natl Acad. Sci USA*, **85**, 2106.

WEISSMAN, B.E., SAXON, P.J., PASQUALE, S.R., JONES, G.R., GEISER, A.G. & STANBRIDGE, E.J. (1987). Introduction of a normal human chromosome 11 into a Wilms' tumor cell line controls its tumorigenic expression. *Science*, **236**, 175.

YOKOTA, J., WADA, M., SHIMOSATO, Y., TERADA. M. & SUGIMURA, T. (1987). Loss of heterozygosity on chromosomes 3, 13, and 17 in small-cell carcinoma and on chromosome 3 in adenocarcinoma of the lung. *Proc. Natl Acad. Sci. USA*, **84**, 9252.

YUNIS, J.J. & RAMSAY, N. (1978). Retinoblastoma and subband deletion of chromosome 13. *Am. J. Dis. Child.*, **132**, 161.

ZBAR, B., BRAUCH, H., TALMADGE, C. & LINEHAN, M. (1987). Loss of alleles of loci on the short arm of chromosome 3 in renal cell carcinoma. *Nature*, **327**, 723.